# Supplementary figures and images for: Early Introduction of Plant Polysaccharides Drives the Establishment of Rabbit Gut Bacterial Ecosystems and the Acquisition of Microbial Functions
Source: mSystems. 2022 Jun 8;7(3):e00243-22. doi: 10.1128/msystems.00243-22 (PMC9239267; doi:10.1128/msystems.00243-22)

**Supplemental Figure S2**

|  | 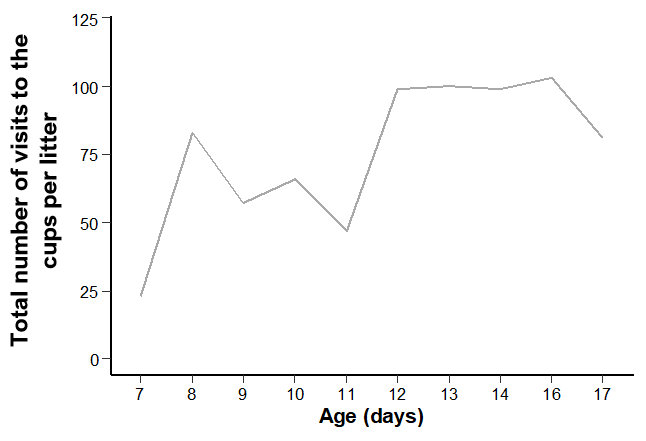 |
| --- | --- |

Supplement: FIG S2 [file msystems.00243-22-s0002.docx]

**Supplemental Figure S4**:

| **A** |
| --- |
| **B**  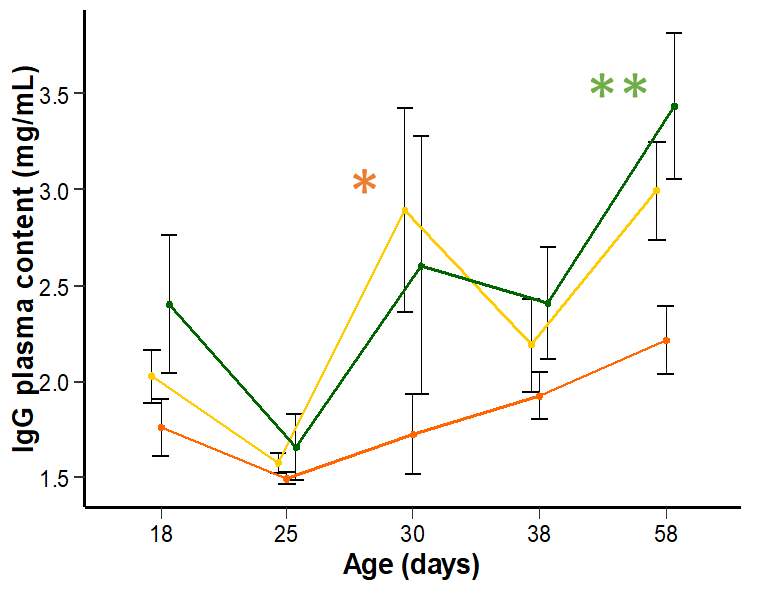 |
| **C**  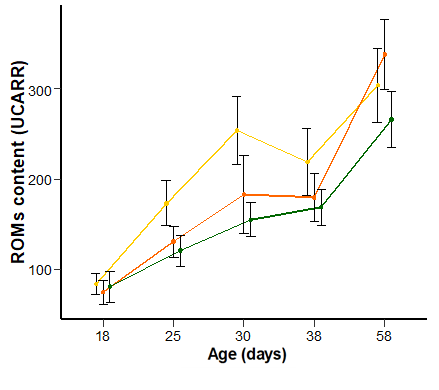 |

Supplement: FIG S4 [file msystems.00243-22-s0004.docx]

**Supplemental Figure S5**:

| **A**  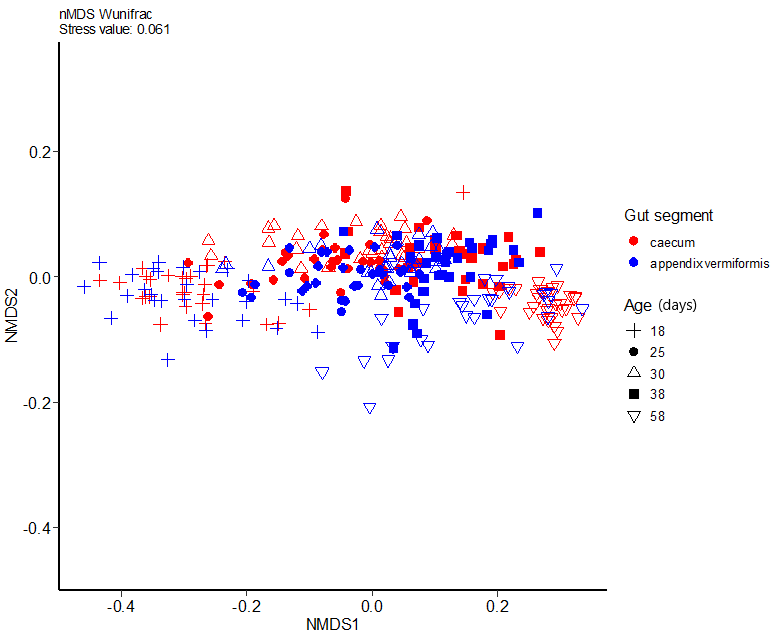 |
| --- |
| **B** |

| **C**  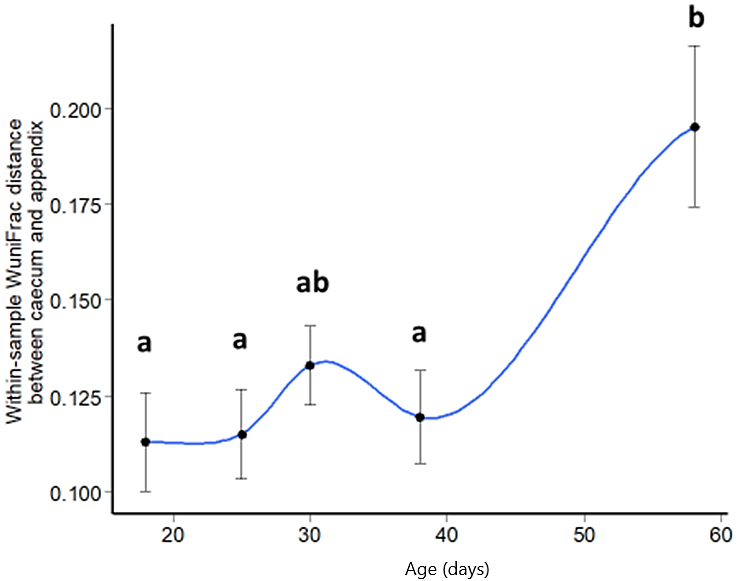 |
| --- |

Supplement: FIG S5 [file msystems.00243-22-s0005.docx]

**Supplemental Figure S6**:

**
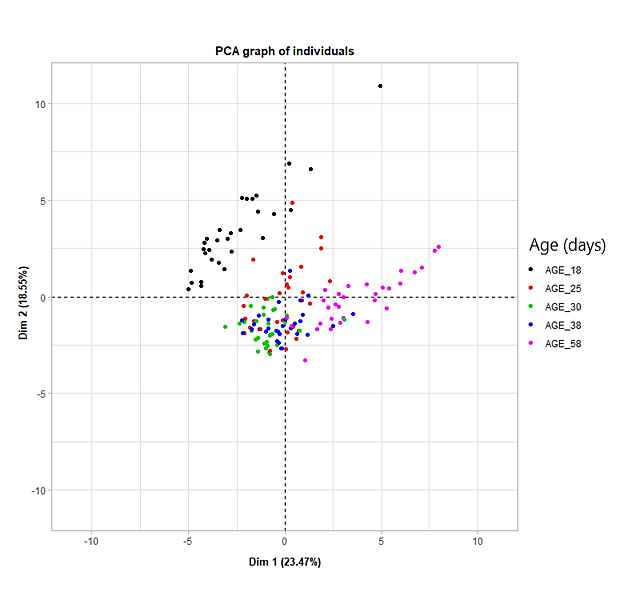
**

Supplement: FIG S6 [file msystems.00243-22-s0006.docx]
