# Supplementary material for: Early Introduction of Plant Polysaccharides Drives the Establishment of Rabbit Gut Bacterial Ecosystems and the Acquisition of Microbial Functions
Source: mSystems. 2022 Jun 8;7(3):e00243-22. doi: 10.1128/msystems.00243-22 (PMC9239267; doi:10.1128/msystems.00243-22)
Supplement: FIG S3 [file msystems.00243-22-s0003.docx]

**Supplemental Figure S3**

| **A**  **** | **B**   |
| --- | --- |
